# Field-based measurement tools to distinguish clonal *Typha* taxa and estimate biomass: a resource for conservation and restoration

**DOI:** 10.3389/fpls.2024.1348144

**Published:** 2024-03-12

**Authors:** Brian M. Ohsowski, Cassidy Redding, Pamela Geddes, Shane C. Lishawa

**Affiliations:** ^1^ School of Environmental Sustainability, Loyola University Chicago, Chicago, IL, United States; ^2^ Department of Biology and Environmental Science Program, Northeastern Illinois University, Chicago, IL, United States

**Keywords:** *Typha* identification, biomass prediction, field assessment, *Typha latifolia*, ecological restoration, *Typha* × *glauca*, conservation

## Abstract

Two species of clonal *Typha* [*T. latifolia* (native) and *T. angustifolia* (exotic)] hybridize to form the highly invasive, heterotic (high vigor) *T. × glauca* in North American wetlands leading to increased primary production, litter accumulation, and biodiversity loss. Conservation of *T. latifolia* has become critical as invasive *Typha* has overwhelmed wetlands. In the field, *Typha* taxa identification is difficult due to subtle differences in morphology, and molecular identification is often unfeasible for managers. Furthermore, improved methods to non-destructively estimate *Typha* biomass is imperative to enhance ecological impact assessments. To address field-based *Typha* ID limitations, our study developed a predictive model from 14 *Typha* characters in 7 northern Michigan wetlands to accurately distinguish *Typha* taxa (n = 33) via linear discriminant analysis (LDA) of molecularly identified specimens. In addition, our study developed a partial least squares regression (PLS) model to predict *Typha* biomass from field collected measurements (n = 75). Results indicate that two field measurements [*Leaf Counts*, *Longest Leaf*] can accurately differentiate the three *Typha* taxa and advanced-generation hybrids. The LDA model had a 100% correct prediction rate of *T. latifolia*. The selected PLS biomass prediction model (*sqrt[Typha Dry Mass] ~ log[Ramet Area at 30 cm] + Inflorescence Presence + Total Ramet Height + sqrt[Organic Matter Depth]*) improved upon existing simple linear regression (SLR) height-to-biomass predictions. The rapid field-based *Typha* identification and biomass assessment tools presented in this study advance targeted management for regional conservation of *T. latifolia* and ecological restoration of wetlands impacted by invasive *Typha* taxa.

## 1 Introduction

Hybridization is a common and important evolutionary mechanism that drives phenotypic diversity, environmental adaptation capacity, and speciation (Mallet, 2005; Goulet et al., 2017). In some cases, plants exhibit heterosis (i.e., hybrid vigor) where hybrid offspring show increased fitness resulting in increased biomass, yield, and root density compared to parental counterparts (Hochholdinger and Baldauf, 2018). Path analysis models suggest plant taxa hybridization propensity at the genus level is significantly correlated with a perennial life cycle, woodiness, and reliance on vegetative reproduction systems (Mitchell et al., 2019). An invasive plant case study also documented that hybridization is often associated with perennial plants exhibiting clonal growth habits as a mechanism leading to fixed heterotic genotypes (Ellstrand and Schierenbeck, 2000). Thus, plant introductions exhibiting hybridization potential with closely related endemic plant populations and clonal growth habits may serve as a precursor to stimulate invasiveness (Ellstrand and Schierenbeck, 2000). In wetland systems, herbaceous wetland plants with clonal growth habits are common among the most highly invasive taxa (Galatowitsch et al., 1999; Zedler and Kercher, 2004).

In North America, two species within the clonal *Typha* (cattail) genus [native *T. latifolia*, non-native *T. angustifolia* (Ciotir et al., 2013)] have hybridized to form *T. × glauca* (Godron) (Smith, 1967). In wetlands, *T. × glauca* exhibits heterosis which typically results in more productive, taller, and faster growing clones that become more dominant compared to either parent species (Zapfe and Freeland, 2015; Bansal et al., 2019). Backcrossing and advanced-generation hybrids are also common (Travis et al., 2010; Kirk et al., 2011; Freeland et al., 2013; Geddes et al., 2021), complicating *Typha* genetic identity in the region (hereafter we will refer to all taxa as *Typha* unless otherwise specified).

The complicated genetics of *Typha* presents a problem for both the management of invasive *Typha* and the conservation of native *T. latifolia.* In the Great Lakes, *Typha* taxa classified as invasive [*T. × glauca* and *T. angustifolia*; hereafter, “invasive *Typha*”] are dominant in more than 13% of the total area of ecologically critical coastal wetland ecosystems (Carson et al., 2018). Along with its continued spread, management of invasive *Typha* has increasingly become a restoration priority (Bansal et al., 2019). Conservation of *T. latifolia* has simultaneously become imperative, due to increased dominance by invasive taxa, hybridization, and backcrossing of hybrids to *T. latifolia* (Pieper et al., 2017; Bansal et al., 2019; Geddes et al., 2021), which could result in extinction by demographic or genetic swamping (Todesco et al., 2016).

Unfortunately, field identification of the three taxa and advanced-generation hybrids using standard morphological characters (e.g., leaf width, gap between inflorescences) can be unreliable due to wide trait variability (Geddes et al., 2021). Molecular methods to identify *Typha* taxa may be impractical, if not entirely unfeasible, for many field practitioners managing invasive species populations and practicing conservation. Although the cost of molecular methods has been decreasing due to technological advancements, application of these techniques is still unrealistic for many practitioners (Hauser and Seeb, 2008; Sagarin et al., 2009). Thus, identifying field morphological characteristics that allow for the accurate differentiation of *Typha* is critical to advance the conservation of *T. latifolia* and the continued management of invasive *Typha*.

Invasive *Typha* taxa are associated with a range of ecological impacts to wetlands. They tend to thrive in wetlands with anthropogenically disturbed hydrology (Boers and Zedler, 2008; Hall and Zedler, 2010; Bunbury-Blanchette et al., 2015). They also outcompete native sedge species (i.e., *Carex stricta*, *C. lacustris*, *C. lasiocarpa*) in wetlands experiencing nutrient enrichment (Woo and Zedler, 2002). Furthermore, invasive *Typha* magnifies nutrient availability by increasing sediment retention (Horppila and Nurminen, 2001) and enhancing internal nutrient cycling (Currie et al., 2014), thus compounding the effects of nutrient enrichment. Invasive *Typha* forms monodominant stands in wetlands by outcompeting native plants and creating a thick layer of slowly decomposing leaf litter (Larkin et al., 2012). Further, they can more than double annual productivity in invaded wetlands (Woo and Zedler, 2002; Angeloni et al., 2006). Once established, invasive *Typha* reduces biodiversity and productivity of native plants (Tuchman et al., 2009), fishes (Schrank and Lishawa, 2019), and aquatic macroinvertebrates (Lawrence et al., 2016).

Accurate and stable methods to estimate productivity are necessary when quantifying metrics of plant dominance, population change, and drivers of biodiversity loss in invasion research (Crystal-Ornelas and Lockwood, 2020). Increased prediction accuracy is also highly desirable when integrating plot-level results (e.g., plant stock concentrations of nutrients, carbon, and heavy metals) across biological scales. Furthermore, plant biomass analyses can confirm and calibrate remote sensing estimates to improve model development for ecological management (Vaz et al., 2018).

Simple linear regression (SLR) standard curves of height-to-biomass have been used to non-destructively predict *Typha* biomass from field traits (Lishawa et al., 2015). Allometric equations are commonly used to non-destructively estimate biomass from forest systems (Henry et al., 2013), but tend to be less robust for herbaceous species with varied morphology and large environmental gradients (Niklas and Enquist, 2002; Pottier and Jabot, 2017). A two-step approach employing Bayesian information criterion (BIC) model selection of plant traits followed by multivariate partial least squares regression (PLS) modeling can produce highly accurate biomass predictions (Ohsowski et al., 2016). This PLS method avoids the pitfalls of excessive destructive sampling, accounts for collinearity among predictor variables, and can employ categorical and continuous data (Ohsowski et al., 2016).

Our study developed new methods that use easily quantified field measurements to accurately identify *Typha* taxa and accurately quantify *Typha* biomass to the benefit of conservation and ecological restoration. Our specific objectives developed prediction models that selected simple field measurements to: 1) accurately classify *Typha* taxa determined by diagnostic microsatellite markers using linear discriminant analysis, and 2) improve *Typha* taxa biomass assessment using BIC model selection and PLS. Additionally, we compared historically employed height-to-biomass SLR model predictions with PLS prediction.

## 2 Materials and methods

### 2.1 Study site selection and experimental design

**Figure 1:**
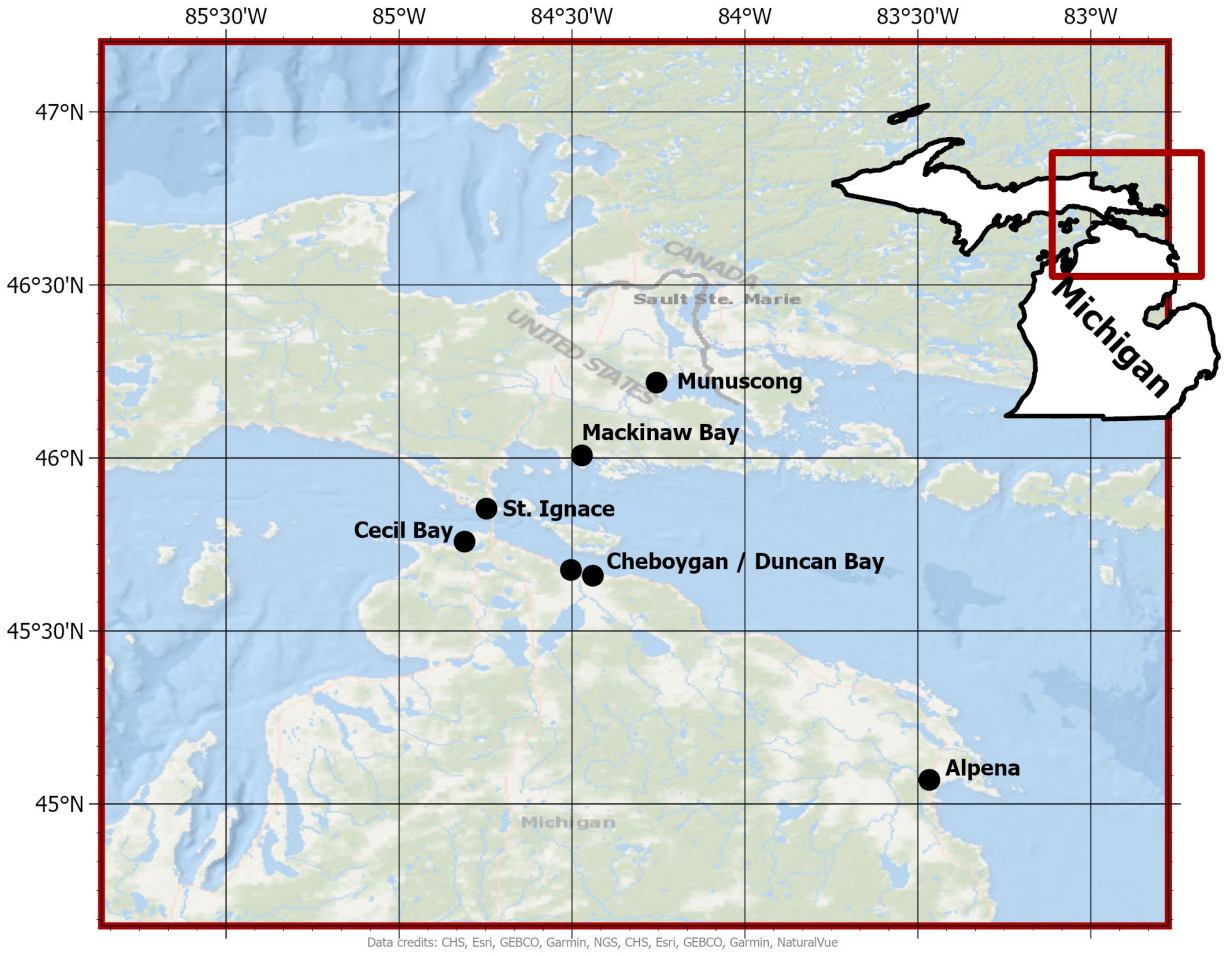
Wetland sites where *Typha* were sampled in northern Michigan (U.S.A.). Black dots represent the geographic center of each wetland site selected for the study.

In July 2021, we identified seven wetlands in northern Michigan (U.S.A.) to conduct the study [eastern Upper Peninsula (3 sites); northern Lower Peninsula (4 sites)]. Six of the seven wetland sites are classified as Great Lakes coastal wetlands (Munuscong Marsh, Mackinaw Bay, St. Ignace Marsh, Cecil Bay, Cheboygan Marsh, and Duncan Bay) and the remaining site was an inland emergent marsh (Alpena Wildlife Sanctuary) (**Figure 1**). All wetlands in the study had *Typha* stands within emergent vegetation zones.

In each wetland, we established a minimum of one continuous transect through the geographic center of established *Typha* stands. In two expansive wetlands with varied water levels and lake exposures (Cheboygan Marsh and St. Ignace Marsh), we increased the number of transects to 3 to capture environmental heterogeneity, resulting in 11 transects total. Nine of 11 transects had a standardized design to include 7 plots (1 m^2^ quadrats) equidistant along a varied transect length depending upon stand extent. The remaining 2 stands were small (< 10 m diameter) but included in the study because we identified the plants as likely *T. latifolia* based on morphology (wide leaves and no separation between staminate and pistillate inflorescences) (Voss and Reznicek, 2012).

### 2.2 Field data collection: morphological characteristics

**Figure 2:**
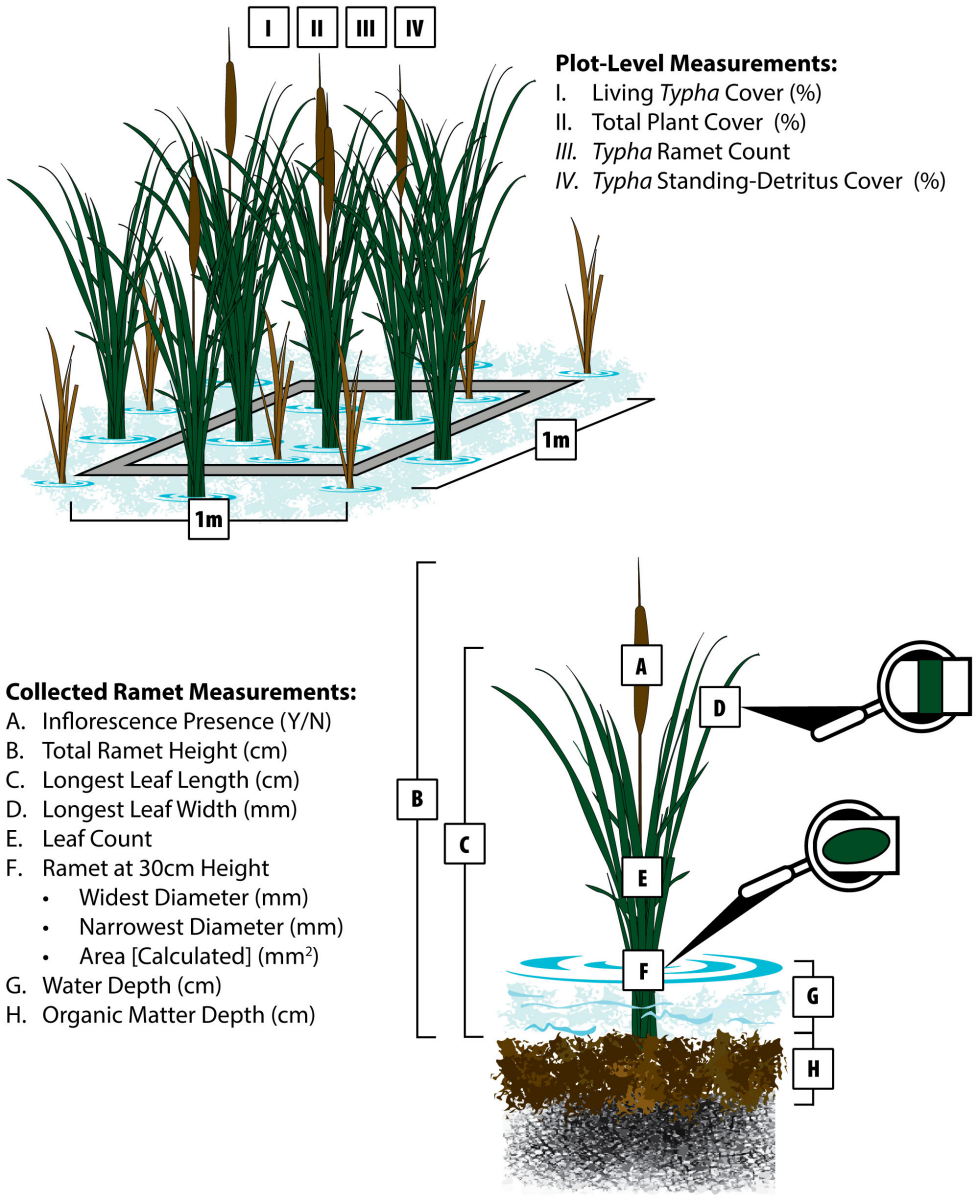
Field measurements collected at each plot (1.0 m^2^ quadrat) and the centermost identified *Typha* ramet. Units are given in parentheses next to each variable listed. Variables (I.-IV.) were collected at the plot scale to assess areal cover and *Typha* ramet density. Plant morphological measurements **(A–F)** and environmental variables **(G, H)** were collected from the centermost *Typha* ramet that was subsequently collected for dry mass and genetic analysis.

At each plot, we visually estimated areal coverage (< 1 – 100%) for plant community living vegetation, *Typha* living vegetation, and *Typha* standing-detritus above the water surface at all plots (**Figures 2I–IV**). We also collected total *Typha* ramet count in each plot to estimate ramet density. Water depth was estimated from the organic matter surface to water surface (**Figure 2G**). Organic matter depth was collected by firmly pushing a graduated PVC pole (1.9 cm diameter) through the decomposed organic layer until contacting mineral sediment (**Figure 2H**).

Following plot-level estimations, we unbiasedly selected the centermost *Typha* ramet to measure variables that potentially predict *Typha* biomass and discriminate *Typha* taxa. Adapted from Ohsowski et al. (2016), we collected the following measurements (**Figures 2A–F**): 1) inflorescence presence (yes/no), 2) total ramet height from organic matter surface (including inflorescence if present), 3) longest leaf length from organic matter surface, 4) maximum leaf width on the identified longest leaf (or longest leaf width), 5) ramet green leaf count, 6) widest ramet diameter at 30 cm, and 7) narrowest ramet diameter at 30 cm. We calculated ramet cross-section area at 30 cm assuming an oval: Area = pi ∗ widest cross-section/2 ∗ narrowest cross-section/2. When present, we measured the gap between pistillate and staminate inflorescences. Following all field measurements, each selected ramet was collected, dried at 60° C, and weighed.

### 2.3 Field data collection: molecular analysis

We established *a priori Typha* leaf tissue collection for molecular analysis from three non-adjacent plots per transect to minimize the probability of collecting clones; for the 2 small stands, tissue was collected from each plot (total replication = 33). Green leaf tissue (length = 10 cm) from the centermost ramet was clipped, bagged, and stored on ice (Geddes et al., 2021). Each leaf sample was then flash frozen in liquid nitrogen in the lab and stored in a cryogenic freezer (-80° C) until molecular analysis. The collected leaf tissue sample wet mass was converted to dry mass and added to total *Typha* dry mass described in Section 2.2.

### 2.4 Molecular analysis

Frozen *Typha* leaf tissue samples were ground with dry ice followed by DNA extraction using Qiagen DNEasy Plant kits. We selected six diagnostic microsatellite markers [TA 3, TA 5, TA 8, and TA 16 (Tsyusko-Omeltchenko et al., 2003), and TM 4 and TM 11 (Csencsics et al., 2010)], shown to be accurate in distinguishing *Typha* species, backcrosses, and advanced-generation hybrids (Geddes et al., 2021 and references therein). PCR amplification of microsatellite primers was accomplished following established protocols (Geddes et al., 2021) using 2-step PCR (Schuelke, 2000), after which a 1.4% agarose gel electrophoresis confirmed successful microsatellite amplification.

We performed microsatellite analyses on a Beckman Coulter gene sequencer with fragment sizing (400 bp ladder), scoring, and microsatellite interpretation analyzed using Beckman Coulter software. Following microsatellite scoring, each of the six microsatellite markers per tissue sample were separated into one of four molecular ID classes: *T. latifolia, T. angustifolia, T. × glauca*, or advanced-generation hybrid (AGH). A sample was categorized as an F1 hybrid (i.e., *T. × glauca*) if one allele from each parental species (*T. latifolia* and *T. angustifolia*) was present. The final taxonomic classification used for statistical analyses was determined when at least 5 of 6 diagnostic microsatellite markers agreed in the molecular ID classification. If diagnostic microsatellite markers did not meet the minimum 5 of 6 consensus among hybrid and/or both parental loci, the sample molecular ID was classified as AGH (Snow et al., 2010; Travis et al., 2010; Travis et al., 2011).

### 2.5 Statistical analysis: *Typha* molecular ID classification

Plot-level *Typha* ramet measurements (**Figure 2**) were used to separate *Typha* molecular ID classes (*T. latifolia, T. angustifolia*, *T. × glauca*, AGH) using linear discriminant analysis (LDA) (replication = 33). *Typha* dry mass and all predictor variables in **Figure 2** were transformed (when required), centered, and scaled to meet statistical assumptions. We used Spearman’s rank correlation coefficients to determine highly collinear variables (r_s_ > 0.90) and remove one of the variable pairs from the analysis. We employed recursive feature elimination with 10-fold cross-validation to select the most relevant class separation variables using the *rfe()* function in R’s *caret* package (Kuhn, 2008; Chen et al., 2020). The selected LDA model was built with the *lda()* function in R’s *MASS* package (Venables and Ripley, 2013). Model and class prediction performance metrics were generated using a confusion matrix in R’s *caret* package (Kuhn, 2008).

We developed an LDA permutation model to estimate test data prediction performance for *Typha* molecular ID classes. For each permutation (n = 1,000), the full dataset (replication = 33) was randomly split into a training data set (replication = 26) to establish an LDA model. The remaining test data (replication = 7) were classified into molecular ID classes by the trained LDA model. We calculated an agreement percentage for predicted within-model training data and external model test data for each iteration as % correctly predicted cases/total cases predicted.

### 2.6 Statistical analysis: biomass prediction

We used plot-level and *Typha* ramet measurements (**Figure 2**) to develop *Typha* dry mass prediction models (replication = 75). Variable standardization, Bayesian Information Criterion (BIC) model selection, and model dry mass prediction workflow followed Ohsowski et al. (2016) and references within. All predictor variable combinations and associated 2^nd^ order polynomial terms were scored with BIC model selection with the *dredge* function in R’s *MuMin* package (Bartoń, 2023). Equivalent multi-variate prediction models (ΔBIC ≤ 2) were averaged using the *model.avg()* function in *MuMin* to provide our selected statistical model employed for *Typha* dry mass prediction.

We trained the selected multi-variate model using partial least squares regression (PLS) (replication = 75) in R’s *pls* package (Liland et al., 2023). Four model components were retained as determined by lowest root mean squared error of cross-validation (RMSECV) calculated from 10-fold cross-validation. For comparison, we developed a simple linear regression (SLR) model for *sqrt[Typha Dry Mass] ~ Total Ramet Height* (replication = 75) using the *lm* function in R’s *base* package (R Core Team, 2023). RMSECV estimates for the SLR model were calculated with 10-fold cross-validation with the *errorest()* function in R’s *ipred* package (Peters and Hothorn, 2023). Similar to Ohsowski et al. (2016), we calculated a simple DIFF term for all *Typha* dry mass predictions with the following formula to assess model prediction performance: DIFF = predicted *Typha* dry mass – reference *Typha* dry mass.

We developed a permutation model for the PLS and SLR models to estimate test data (i.e., external data) prediction performance for *Typha* dry mass, thus assessing model robustness and real-world model applicability. For each permutation (n = 1,000), the full dataset (replication = 75) was randomly split into training data (replication = 63) to develop the PLS and SLR models. The remaining test data (replication = 12) were predicted by the trained PLS and SLR models, back-transformed, and DIFF term was calculated for the resulting permutation model.

## 3 Results

### 3.1 Variable selection: molecular ID classification

We developed a linear discriminant analysis (LDA) to predict *Typha* molecular ID classes: *T. latifolia, T. angustifolia*, *T. × glauca*, and AGH. To meet test assumptions, we dropped three collinear variables from the analysis using Spearman’s rank correlation coefficients: *Longest Leaf Length* (collinear with *Total Ramet Height*, r_s_ = 0.996), *Widest Ramet Diameter at 30 cm* (collinear with *Ramet Area at 30 cm*, r_s_ = 0.974), and *Narrowest Ramet Diameter at 30 cm* (collinear with *Ramet Area at 30 cm*, r_s_ = 0.928). Total ramet height was selected over longest leaf length as this character is a very simple field measurement. We also dropped the widest and narrowest ramet diameter measurements as they were highly collinear with the calculated ramet areas due to formula inclusion. In total, ten variables were used to determine the most parsimonious LDA model via recursive feature elimination with 10-fold cross-validation: *sqrt[Organic Matter Depth], sqrt[Water Depth], sqrt[Living Typha Cover], log[Typha Detritus Cover], sqrt[Leaf Count]*, *sqrt[Longest Leaf Width]*, *sqrt[Longest Leaf Length], sqrt[Typha Ramet Count], Typha Height, log[Ramet Area at 30 cm]*.

We determined the most relevant LDA class separation variables for the final LDA model: *Molecular ID Class* ~ *sqrt[Leaf Count]* + *sqrt[Longest Leaf Width]*. Note that *sqrt[Water Depth]* was retained in the initial selected recursive feature elimination model but removed from this analysis. We determined that including water level measurements may lead to unreliability for future application of the presented model by increasing uncertainty (*see Discussion*).

### 3.2 Diagnostic microsatellite markers: molecular ID classification

Agreement among the six microsatellite markers resulted in four molecular ID classes: *T. latifolia, T. angustifolia*, *T. × glauca*, and AGH. Out of the 33 molecular samples, 66.3% had complete diagnostic microsatellite agreement among all six molecular markers [count]: *T. angustifolia* [3], *T. × glauca* [15], *T. latifolia* [3]. Additionally, 30.3% of the samples had consensus in 5 out of 6 markers resulting in molecular ID classification [count] of *T. angustifolia* [5], *T. × glauca* [3], and *T. latifolia* [2]. Two samples were classified as AGH because the six microsatellite markers were split in the molecular ID: one of the samples had 3 markers identifying it as *T. × glauca* and 3 markers as *T. angustifolia*, while the other sample had 2 markers identifying it as *T. × glauca* and 4 markers as *T. angustifolia*. These two latter samples likely represent backcrosses to one of the parental species (in this case *T. angustifolia*). Given our relatively low sample size for molecular analyses (n = 33), we categorized all hybrids beyond the F1 hybrid as advanced-generation hybrids. Overall, molecular ID analyses prevalence revealed that 15.2% of our samples were classified as *T. latifolia*, 24.2% as *T. angustifolia*, 54.6% as *T. × glauca*, and 6.1% as AGH (**Table 1**).

**Table 1:** Linear discriminant analysis (LDA) model performance metrics by class for training data to separate the four *Typha* molecular ID classes: *Typha angustifolia* [A], Advanced Generation Hybrid [AGH], *Typha × glauca* [G], and *Typha latifolia* [L].

LDA *Typha* Molecular ID by Class				
	A	AGH	G	L
Sensitivity (True Positive Rate)	87.5%	0.0%	77.8%	100.0%
Specificity (True Negative Rate)	80.0%	96.8%	93.3%	100.0%
Prevalence	24.2%	6.1%	54.6%	15.2%
Balanced Accuracy	83.8%	48.4%	85.6%	100.0%
LDA Gravity Centered Means				
Longest Leaf Width (mm)	7.06	8.02	10.56	15.05
Leaf Count	6.35	4.94	7.89	12.98
Raw *Typha* Measurements by Class				
Class Replication	8	2	18	5
Longest Leaf Width (mm) (mean ± 1 sd)	7.09 ± 0.91	8.03 ± 0.04	10.69 ± 2.28	15.10 ± 1.85
Leaf Count (mean ± 1 sd)	6.38 ± 0.92	5.00 ± 1.41	7.94 ± 1.35	13.00 ± 1.00

For the training data statistics, class-based sensitivity, specificity, prevalence, and balanced accuracy are given. LDA derived molecular ID group mean centers of gravity are given on the original measurement scale. In addition, raw data summary statistics for *Typha* morphological measurements and replication are given for comparison to LDA’s mean center of gravity predictions.

### 3.3 Molecular ID class separation

**Figure 3:**
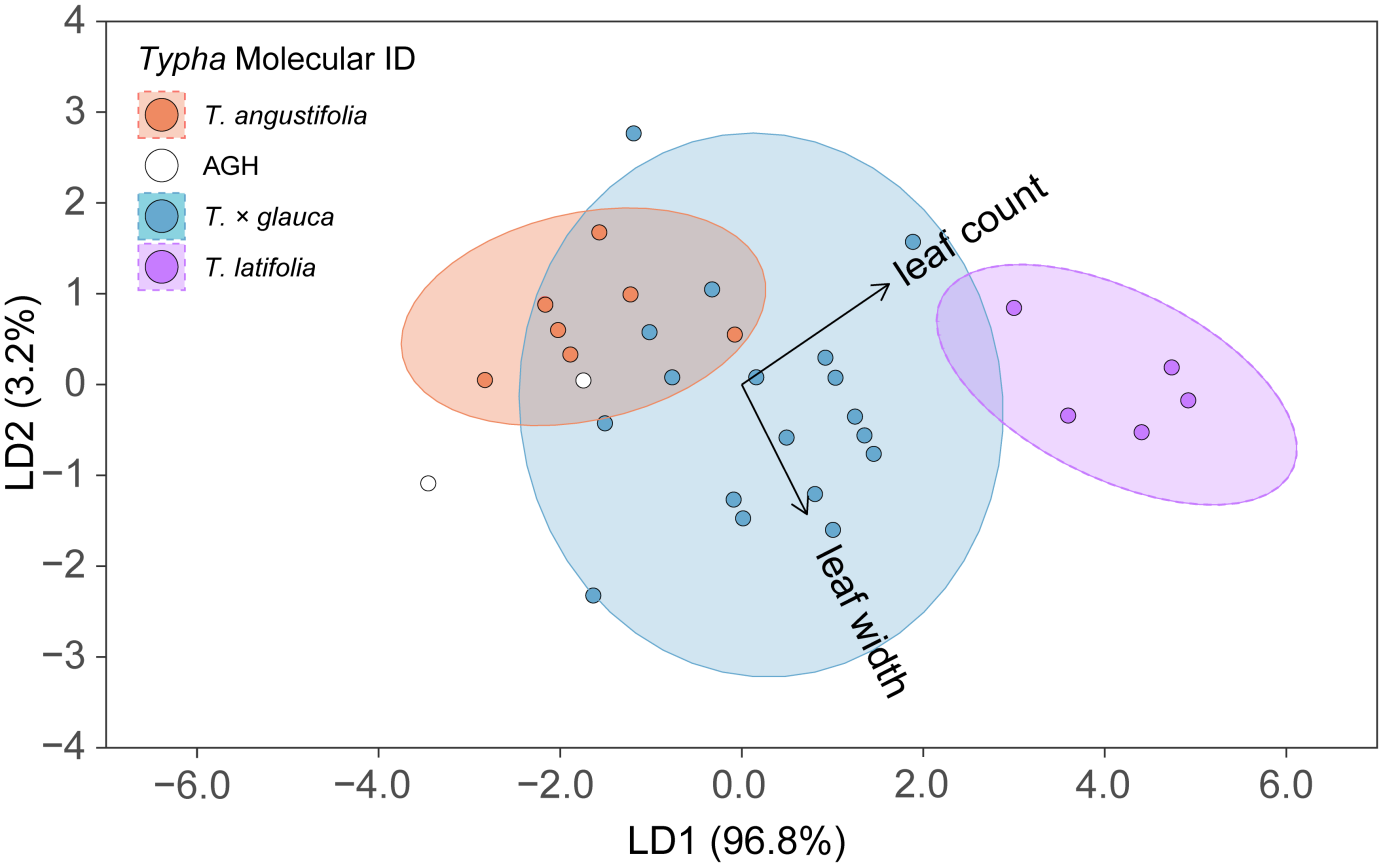
Linear discriminant analysis (LDA) results displaying linear discriminant function 1 (LD1) vs. linear discriminant function 2 (LD2) to separate the four *Typha* molecular ID classes: *Typha angustifolia*, advanced generation hybrid [AGH], *Typha × glauca*, and *Typha latifolia*. LD1 vs. LD2 maximized class separation given with parenthetic values for *Proportion of Trace* describing discriminant function explained variation. Respective ellipses represent 95% confidence intervals for each predicted class. Arrows represent the contribution direction and magnitude of each predictor variable. Final LDA model: *sqrt[Typha Dry Mass] ~ sqrt[Leaf Count] + sqrt[Longest Leaf Width]*. AGH had insufficient data to provide model confidence intervals.

The overall linear discriminant analysis model had high statistical accuracy when predicting the four *Typha* molecular classes. LDA training data confusion matrix statistics revealed high confidence for internal prediction model accuracy [correct % prediction (± 95% CI): 78.8% (61.1%, 91.0%), Kappa = 66.8%]. The LDA model significantly outperformed the no information rate (i.e., null) model (*p* = 0.003) (**Table 2**). The two most descriptive linear discriminant functions (LD1: explained variance 96.8%; LD2: explained variance 3.2%) successfully separated the molecular ID classes driven by the leaf count and longest leaf width variables (**Figure 3**). The 95% *T. angustifolia* confidence intervals more strongly overlapped with *T. × glauca* compared to the clear class separation between *T. × glauca* and *T. latifolia* (**Figure 3**). *T. latifolia* and *T. angustifolia* had no 95% confidence interval overlap indicating clear molecular ID class separation between the two taxa (**Figure 3**). AGH had only two classification instances (6.1% of data set) leading to instability in predicting the class (**Table 1**) and unresolved 95% confidence intervals (**Figure 3**) due to low replication. LDA models revealed 100% accurate classification of *T. latifolia*, 85.6% for *T. × glauca*, and 83.8% for *T. angustifolia* (**Table 1**). Molecular class gravity centered mean measurement values were back-transformed and presented for leaf count and longest leaf width in **Table 1**.

**Table 2:** Overall linear discriminant analysis (LDA) model performance metrics to separate the four *Typha* molecular ID classes.

LDA *Typha* Molecular ID Training Data	
**Response Variable:**	*Typha* Molecular ID
**Predictor Variables:**	sqrt[Leaf Count] + sqrt[Longest Leaf Width]
Correctly Predicted:	78.8%
Correctly Predicted 95% CI:	(61.1%, 91.0%)
P-Value [Acc>NIR]:	0.003
Kappa:	66.8%
LDA *Typha* Molecular ID Permutation Model	
Number of Permutations:	1,000
Training / Test Replication:	n = 26 / n = 7
Training Data (% correct ± 1 sd):	87.9% ± 3.6%
Test Data (% correct ± 1 sd):	78.7% ± 15.3%

LDA training data statistics were extracted from a generated confusion matrix in R’s *caret* package. Permutation model statistics were developed to estimate test data prediction performance for *Typha* molecular ID classes from both training data and test data. An agreement percentage for predicted data for each iteration was calculated as % correctly predicted cases/total cases predicted and permutation results subsequently averaged.

The permutation LDA model result confirmed high prediction agreement for training data and test data of molecular ID classes. For each permutation iteration (n = 1,000), 78.8% of the full data set trained the LDA model to externally predict 21.2% of the test data. The average % correct molecular ID prediction for each permutation iteration confirmed model accuracy for internal training data (mean ± 1 sd % correct: 87.9% ± 3.6%) and test data (mean ± 1 sd % correct: 78.7% ± 15.3%) (**Table 2**).

### 3.4 Variable selection: *Typha* dry mass prediction

We used 14 predictor variables (and associated polynomial terms) for BIC model selection. Model selection resulted in 11 equivalent models that predicted *Typha* dry mass in the study (**Table 3**). Following BIC model averaging, the final PLS prediction model was reduced to 4 predictor variables: *sqrt[Typha Dry Mass] ~ log[Ramet Area at 30 cm] + Inflorescence Presence + Total Ramet Height + sqrt[Organic Matter Depth]*.

**Table 3:** Equivalent BIC selected models (ΔBIC ≤ 2) and associated degrees of freedom (df) generated to predict *Typha* dry mass.

Equivalent *Typha* Dry Mass Prediction Models	df	Δ BIC
Response Variable: sqrt [*Typha* Dry Mass]		
Predictor Variables:		
log[Ramet Area at 30 cm] + Inflorescence Presence + Leaf Count + Longest Leaf Length + sqrt[Organic Matter Depth]	7	0.00
log[Ramet Area at 30 cm + Inflorescence Presence + Longest Leaf Length + sqrt[Organic Matter Depth]	6	0.18
log[Ramet Area at 30 cm + Inflorescence Presence + sqrt[Organic Matter Depth] + Total Ramet Height	6	0.43
log[Ramet Area at 30 cm] + Inflorescence Presence + Leaf Count + sqrt[Organic Matter Depth] + Total Ramet Height	7	0.56
log[Ramet Area at 30 cm] + (log[Ramet Area at 30 cm])^2^ + Inflores- cence Presence + Longest Leaf Length + sqrt[Living *Typha* Cover] + (sqrt Living *Typha* Cover])^2^	8	0.88
log[Ramet Area at 30 cm] + (log[Ramet Area at 30 cm])^2^ + Inflorescence Presence + Longest Leaf Length + sqrt[Organic Matter Depth]	7	1.04
log[Ramet Area at 30 cm + (log[Ramet Area at 30 cm])^2^ + Inflorescence Presence + sqrt[Organic Matter Depth] + Total Ramet Height	7	1.49
log[Ramet Area at 30 cm] + (log[Ramet Area at 30 cm])^2^ + Inflores- cence Presence + Total Ramet Height + sqrt Living *Typha* Cover + (sqrt Living *Typha* Cover])2	8	1.55
log[Ramet Area at 30 cm + Inflorescence Presence + sqrt[Organic Matter Depth]	5	1.81
log[Ramet Area at 30 cm] + (log[Ramet Area at 30 cm])^2^ + Inflorescence Presence + Leaf Count + Longest Leaf Length + sqrt Organic Matter Depth]	8	1.98
log[Ramet Area at 30 cm + Inflorescence Presence + Leaf Count + Longest Leaf Length + sqrt Organic Matter Depth + sqrt[Living *Typha* Cover + (sqrt[Living *Typha* Cover])^2^	9	1.98

Variables were transformed (where indicated) and subsequently centered and scaled (variable mean = 0, variance = 1) prior to BIC selection.

Six of 14 potential predictor variables (and associated polynomial terms) in **Figure 2** were not selected in any equivalent ΔBIC ≤ 2 models: *Typha* standing-detritus cover, widest ramet diameter at 30 cm, narrowest ramet diameter at 30 cm, maximum leaf width on the longest leaf (i.e., longest leaf width), ramet green leaf count, and water depth (**Table 3**). Prevalent variables selected within ΔBIC ≤ 2 models (% occurrence across ΔBIC ≤ 2 models, n = 11) were: ramet area at 30 cm (100%), inflorescence presence (100%), and organic matter depth (81.8%). Longest leaf length (54.5%) and total ramet height (36.3%) were never selected for the same ΔBIC ≤ 2 equivalent model. Although longest leaf length was selected more frequently, total ramet height (36.3%) was chosen as a preferred PLS predictive variable because of the relative ease of collecting ramet height data in the field without compromised predictive power. Curvilinear relationships (i.e., 2^nd^ order polynomial terms) were infrequently included to predict *Typha* dry mass for ramet area at 30 cm and living *Typha* cover (**Table 3**). Polynomial terms were not influential after BIC model averaging.

### 3.5 PLS and SLR *Typha* dry mass prediction

**Figure 4:**
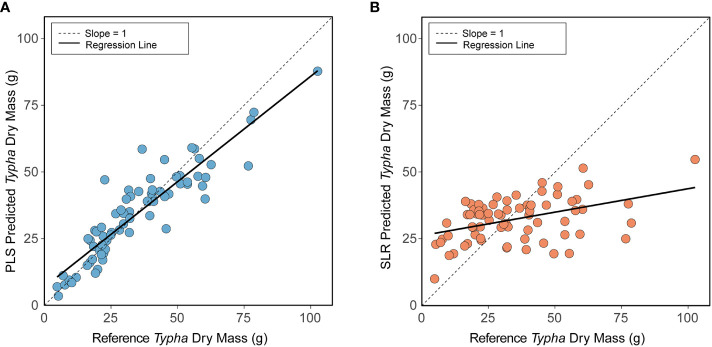
Partial least squares regression (PLS) **(A)** and simple linear regression (SLR) **(B)** model results for predicted *Typha* dry mass (training data) vs. reference *Typha* dry mass. Panel **(A)** Training data predicted from final PLS model generated from BIC model selection*: sqrt[Typha Dry Mass] ~ log[Ramet Area at 30 cm] + Inflorescence Presence + Total Ramet Height + sqrt[Organic Sediment Depth]*. Panel **(B)** Training data predicted from final SLR prediction model: *sqrt[Typha Dry Mass] ~ Total Ramet Height.* In both panels, the dashed line (—) represents a slope = 1 indicating a perfect prediction between reference *Typha* dry mass and predicted *Typha* dry mass. The solid black line (**—**) represents the actual best fit regression line between predicted *Typha* dry mass and reference *Typha* dry mass.

*Typha* dry mass descriptive statistics (min: 4.78 g; max: 102.62 g; mean ± 1 sd: 34.52 g ± 19.22 g) successfully characterized plant population size class ranges encountered in the study’s wetlands. To this end, the multi-variate partial least squares regression (PLS) *Typha* dry mass prediction model vastly outperformed the simple linear regression (SLR) prediction model developed for *sqrt[Typha Dry Mass] ~ Total Ramet Height*. The trained PLS prediction model [Root mean squared error of cross validation (RMSECV): 0.47 g, explained variance: 85.01%, replication = 75] had higher accuracy and precision when validating model performance compared to the trained SLR model [RMSECV: 2.27 g, explained variance: 18.38%, replication = 75] (**Table 4**). Thus, utilizing the selected PLS model instead of the simple SLR resulted in greater accuracy in predicting *Typha* dry mass (**Figure 4**). For clarity in **Figure 4**, the slope = 1 reference line indicates a perfect prediction between predicted and reference dry mass. In **Figure 4A**, the linear regression of predicted PLS *Typha* dry mass ~ reference *Typha* dry mass (*p* < 0.001, R^2^ = 0.832) had high agreement and low unexplained variation with slope = 1 and the regression fit. Compared to slope = 1, the PLS model slightly underpredicted the reference dry mass of *Typha* within the higher biomass ranges. In contrast (**Figure 4B**), the linear regression of predicted SLR *Typha* dry mass ~ reference *Typha* dry mass (*p* < 0.001, R^2^ = 0.180) displayed strong skew, low agreement, and high unexplained variation when compared to slope = 1 and the regression fit. The SLR vastly underpredicted *Typha* dry mass as reference dry mass increased, resulting in lower model confidence compared to the PLS model.

**Table 4:** Partial least squares regression (PLS) and simple linear regression (SLR) model performance metrics for *Typha* dry mass predictions.

PLS *Typha* Dry Mass Training Data Statistics	
**Response Variable:**	sqrt [*Typha* Dry Mass]
**Predictor Variables:**	log[Ramet Area at 30 cm] + Inflorescence Presence + sqrt[Organic Matter Depth] + Total Ramet Height
PLS Components:	4
Cross-Validation Segments:	10
RMSECV *Typha* Dry Mass:	0.47 g
Explained Variance:	85.01%
SLR *Typha* Dry Mass Training Data Statistics	
**Response Variable:**	sqrt [*Typha* Dry Mass)
**Predictor Variable:**	Total Ramet Height
Cross-Validation Segments:	10
RMSECV *Typha* Dry Mass:	2.27 g
Explained Variance:	18.38%
*Typha* Dry Mass Test Data Statistics	
Number of Permutations:	1,000
Training/Test Replication:	n = 63 / n = 12
PLS DIFF (mean ± 1 sd):	-0.53 g ± 8.70 g
SLR DIFF (mean ± 1 sd):	-2.37 g ± 18.0 g

Training data statistics for PLS and SLR models present predictor variable(s), components selection (PLS only), *k*-fold cross-validation segments, root mean square error of cross-validation (RMSECV), and explained model variance. Test data statistics are given for permutation models results to estimate accuracy of external data predictions. Permutation results are given by mean ± 1 standard deviation of: DIFF = [*predicted Typha dry mass – reference Typha dry mass]*. All presented *Typha* dry mass results were back-transformed to show original data collection scale.

The permutation model results further confirmed superior PLS model performance compared to SLR model performance. For each permutation iteration (n = 1,000), 84.0% of the full data set trained the respective PLS and SLR model to externally predict 16% of the test data. Calculated DIFF confirmed high PLS model accuracy for test data (mean DIFF ± 1 sd: -0.53 g ± 8.70 g) compared to the more highly variable SLR model test data (mean DIFF ± 1 sd: -2.37 g ± 18.00 g).

## 4 Discussion

Our study emphasized field applicability from simple aboveground *Typha* morphological measurements to support rapid ecological management decisions for wetland plant conservation and restoration. Contextually, field data collected in this study occurred during peak growing season in northern Michigan (July-August). Thus, our predictive models will have widest applicability to fully mature *Typha* ramets prior to senescence. Overall, we are confident that both of our developed techniques can be employed with high precision and accuracy to generate reliable data for researchers and land managers combatting invasive *Typha* populations and implementing conservation strategies to protect *T. latifolia* in North America.

Molecular marker results indicate that hybridization is common across the study region and that introgression (e.g., hybridization beyond the F1 hybrids) may not be as prevalent in this study area compared with others (e.g., Geddes et al., 2021 and references therein). Specifically, we only identified advanced-generation hybrids twice across all samples. However, we contend that the discrepancy in classified molecular cases was not necessarily a major limitation of this study. This study’s design did not prioritize quantification of occurrence frequency of *Typha* in the region as we did not specifically target equal population sizes with the intent for balanced replication or a comprehensive wetland selection protocol of all regional extant *Typha* stands. Thus, direct comparisons with prior studies that address prevalence or occurrence frequency of *Typha* taxa in North America should be avoided.

In our sampled wetlands, LDA model selection revealed that simple field measurements exhibited good taxa separation. Morphological measurements of *T. × glauca* fell in between *T. angustifolia* and *T. latifolia* in both LDA mean center of gravity and raw data summary statistics (**Table 1**). These results agree with Snow et al. (2010) who found that microsatellite markers sorted samples by measured traits into three distinct clusters represented by *T. latifolia*, *T. × glauca*, an*d T. angustifolia*. Similar to our study, the results of Snow et al. (2010) located *T. × glauca* in the middle of the parental species clusters. Furthermore, our results agree with those of Kirk et al. (2011) and those of Kuehn and White (1999) who found that principal component analysis (PCA) of microsatellite markers and discriminant analyses of randomly amplified polymorphic DNA (RAPD) markers, respectively, categorized *Typha* samples into three distinct clusters (*T. latifolia*, *T. × glauca*, and *T. angustifolia*) by measured plant traits.

We assert that our capacity to identify *T. latifolia* is timely and crucial when detecting and distinguishing the increasingly rare *T. latifolia* from invasive *Typha*. Our study successfully showed that the molecular ID of *T. latifolia* was strongly separated from the remaining *Typha* taxa with slight 95% confidence interval overlap with *T. × glauca* and no overlap with *T. angustifolia* (**Figure 3**). Our method to accurately identify *T. latifolia* with two measurements will allow field biologists to differentiate populations of the native species quickly and accurately from the invasive taxa to improve conservation efforts.

In our study, LDA longest leaf width mean center of gravity for *T. latifolia* (15.05 mm) was reliably distinguished from longest leaf width for *T. angustifolia* (6.35 mm) and *T. × glauca* (10.56 mm) (**Figure 2**; **Table 1**). In Snow et al. (2010), cluster classification corresponded well with several plant field measurements that included: log(leaf length/leaf width), length of gap between inflorescences, inflorescence length, and stem diameter. In confirmation with our results, a measurement metric including leaf width [i.e., log(leaf length/leaf width)] was most useful in distinguishing between parental species and the F1 hybrid (Snow et al., 2010). In contrast, our LDA model did not select a diagnostic stem measurement (e.g., ramet area at 30 cm) as a strong predictor of class separation. In Kirk et al. (2011), *Typha* taxa clustering was also significantly driven by leaf width measurements from a random subset of selected leaves. Given the high variability of leaf width within a particular ramet based on position, size, or age, our selection of a ramet’s longest leaf width will increase measurement consistency to yield a more robust metric. Lastly, in Kuehn and White (1999), cluster classification of the three *Typha* taxa corresponded with stigma width, length of inflorescence spike, gap between inflorescences, leaf width, and inflorescence width. However, they concluded that no single character or sets of characters were diagnostic due to considerable overlap among parental species and the hybrid. In addition, 4 of 5 characters used by Kuehn and White (1999) relied on the presence of inflorescences and included more complex, microscope-assisted measurements of stigma widths.

Our second LDA selected character (leaf count), is, to our knowledge, a novel measurement not previously identified as a *Typha* taxa classification trait. In our study, LDA leaf count mean center of gravity for *T. latifolia* (12.98 leaves) far surpassed leaf count for *T. angustifolia* (6.35 leaves) and *T. × glauca* (7.89 leaves) suggesting a simple measurement metric can be used in combination with longest leaf width to improve classification prediction (**Figure 2**; **Table 1**). Wasko et al. (2022) employed mean leaf-apex angle measured for *Typha* ramet leaves (range: 2-9 leaves; mean: 5.3 leaves per ramet) to successfully match *Typha* ID to microsatellite markers. In contrast to leaf count, the mean leaf-apex angle metric in Wasko et al. (2022) requires a labor investment in the field. As our study did not include mean leaf-apex angle, future models could include this trait to determine if its contribution greatly improves predictive class separation.

We still argue that field-based characters related to *Typha* inflorescence measurements are extremely helpful in taxa differentiation, specifically the gap between the staminate and pistillate inflorescences. Yet, we caution that relying on inflorescence trait measurements may be problematic. *Typha* clonal vegetative growth, which allows spread via rhizomes, can be highly plastic regarding inflorescence production (Grace and Wetzel, 1982). In our current study, this was evidenced by the fact that 73% (55 of 75) of the randomly collected centermost ramets lacked an inflorescence. Wasko et al. (2022) also found that less than 50% (22 of 45) of their sampled *Typha* ramets had inflorescences. Furthermore, large-scale management focused on aboveground biomass removal via harvesting has resulted in stark reduction in inflorescence frequency in subsequent years post-harvest. For instance, following two consecutive years of invasive *Typha* harvest at Shiawassee National Wildlife Refuge (MI, USA), 0.22% of *Typha* ramets produced an inflorescence, compared with 16.66% of *Typha* ramets in unharvested control plots (Lishawa et al., 2020). Our field-based *Typha* measurements investigated in this study relied solely upon vegetative growth characteristics, thus providing a wider applicability for land managers and researchers in the field.

As noted in the results, the water depth variable in the LDA model improved *Typha* class separation accuracy by approximately 9.1%. We removed this variable as a potential predictor variable as 6 of the 7 sampled wetlands were classified as Great Lakes coastal wetlands. As water levels fluctuate considerably in the Great Lakes (Gronewold and Rood, 2019), small predictive gains for variable retention were determined to not outweigh the potentially erroneous predictive conclusions. In Great Lakes coastal wetland systems, daily water level ranges exceeding 20 cm are common due to seiche events (Trebitz, 2006). The static LDA predictive model assumes stability in water levels to separate the molecular ID classes. Future model improvement may consider including water level measurement in less dynamic, inland wetlands and/or with greater sampling breadth of *T. latifolia* populations. As *T. latifolia* populations are increasingly rare in the region, population sampling was limited to two small *T. latifolia* stands (< 10 m diameter) in this study. Summary statistics suggest that water level for *T. latifolia* (mean ± 1 sd: 7.90 cm ± 5.81 cm) may be a viable indicator in future studies but remains unreliable in this study due to high variation in *T. × glauca* and *T. angustifolia* (mean ± 1 sd: 31.03 cm ± 16.93 cm; 63.31 cm ± 40.05 cm, respectively). Furthermore, evidence from Lake Ontario wetlands with co-occurring *Typha* taxa suggests that the three dominant taxa do not tend to sort along a water depth gradient, but instead occupy similar habitats (McKenzie-Gopsill et al., 2012). Taken together, this evidence provided additional justification for dropping water depth from our model.

Variables selected for our PLS equations incorporated total ramet height, organic matter depth, inflorescence presence, and ramet area at 30 cm. The resulting PLS model was 85.01% accurate at predicting *Typha* dry mass training data, thus improving upon published allometric equations for *Typha* (Lishawa et al., 2015). Furthermore, our PLS model was robust to test (i.e., external) data predictions leading to high confidence in our model prediction applicability (**Table 4**, PLS DIFF: 0.53 g ± 8.70 g). Comparatively, SLR models solely using total ramet height performed poorly when predicting test data *Typha* dry mass (**Table 4**, PLS DIFF: -2.37 g ± 18.0 g). Similar to Ohsowski et al. (2016), model predictive performance with multi-variate traits vastly improved both precision and accuracy for training and test data predictions. Unsurprisingly, plant height has been used as a variable to create plant biomass predictive standard curves or as a proxy for plant biomass (Catchpole and Wheeler, 1992). Our PLS model highlights that the sole use of plant height measurements contributes to a high level of model uncertainty, especially at higher biomass values for *Typha* specifically. Our study further affirms that plant height does provide predictive power when used in conjunction with multi-variate model predictors.

Interestingly, our proposed PLS model provides researchers with additional novel morphological measurements to accurately predict *Typha* biomass. In this context, ramet area at 30 cm was a consistently selected variable to improve *Typha* dry mass prediction. Ramet area at 30 cm alludes to the thickness and shape of the culm calculated from widest ramet diameter and narrowest ramet diameter. In their molecular ID study, Snow et al. (2010) found that stem diameter helped explain the separation of the parental species and the F1 hybrid. Here, we provide evidence that ramet area is also useful in explaining predicted *Typha* dry mass. At first glance, this trait may seem challenging to measure in the field. In our experience, integrating ramet area measurement can be accomplished with common fieldwork tools such as calipers or a flexible measuring tape. We assert that including this variable is essential despite minor increases in time and labor since the multi-variate PLS model substantially increased explained variance and test data prediction precision.

Another unexpected, but reasonable, predictor of *Typha* dry mass was organic matter depth. Organic depth has been correlated with measures of *Typha* dominance. For example, in 14 Great Lakes coastal wetlands in our project region organic matter depth was more than 3-times greater and sediment ammonium was over 10-times greater where *Typha* was present (Lishawa et al., 2010). Further, *Typha* ramet density was positively correlated with organic matter depth (Lishawa et al., 2010). Organic sediments in these freshwater coastal systems are likely a strong proxy for sediment nutrient availability. *Typha* has been shown to increase sediment retention (Horppila and Nurminen, 2001), thereby creating a nutrient retention positive feedback. Corroborating these results, reviewed research indicates that roots of stoloniferous and rhizomatous species clones proliferate rapidly under conditions of increased nutrient resource availability (de Kroons and Hutchings, 1995). Thus, organic matter depth should be expected to drive plant vigor.

In conclusion, our results will benefit the work of land managers and conservation biologists by enabling the rapid identification of *Typha* taxa with minimal effort in the field. Furthermore, our biomass prediction models will lend greater confidence in non-destructive field-based measurements to improve scaled-up plot level data to the landscape level. As intended, we are confident that this study will help North American land managers parse subtle morphological trait variation in *Typha*, enhancing wetland conservation and ecological restoration efforts.

## Data availability statement

The original contributions presented in the study are publicly available. This data can be found here: https://osf.io/74yvr.

## Author contributions

BO: Conceptualization, Data curation, Formal analysis, Funding acquisition, Methodology, Project administration, Writing – original draft, Writing – review & editing. CR: Data curation, Methodology, Writing – original draft. PG: Conceptualization, Data curation, Formal analysis, Funding acquisition, Methodology, Writing – original draft, Writing – review & editing. SL: Conceptualization, Data curation, Formal analysis, Funding acquisition, Investigation, Methodology, Writing – original draft, Writing – review & editing.
